# ^18^F-Fluorodeoxyglucose positron emission tomography for detection of acute cellular rejection after heart transplantation: A single–center retrospective study

**DOI:** 10.1016/j.jhlto.2026.100526

**Published:** 2026-02-24

**Authors:** Tomoaki Yoshitake, Takeo Fujino, Toru Hashimoto, Shoei Yamamoto, Kei Ikuta, Tomoyasu Suenaga, Kayo Misumi, Keisuke Shinohara, Shouji Matsushima, Yoshiyuki Kitamura, Takuro Isoda, Shingo Baba, Shintaro Kinugawa, Kousei Ishigami, Akira Shiose, Kohtaro Abe

**Affiliations:** a Department of Cardiovascular Medicine, Faculty of Medical Sciences, Kyushu University, Fukuoka, Japan; b Department of Advanced Cardiopulmonary Failure, Faculty of Medical Sciences, Kyushu University, Fukuoka, Japan; c Department of Clinical Radiology, Faculty of Medical Sciences, Kyushu University, Fukuoka, Japan; d Department of Health Sciences, Graduate School of Medical Sciences, Kyushu University, Fukuoka, Japan; e Department of Cardiovascular Medicine, NHO Fukuoka National Hospital, Fukuoka, Japan; f Department of Cardiovascular Surgery, Faculty of Medical Sciences, Kyushu University, Fukuoka, Japan

**Keywords:** heart transplantation, heart failure, rejection, acute cellular rejection, ^18^F-FDG PET/CT

## Abstract

**Background:**

Although histopathological evaluation of myocardium is essential for detecting both acute cellular rejection (ACR) and antibody-mediated rejection (AMR) following heart transplantation, indeterminate results and complication risks associated with repeated endomyocardial biopsies remain serious problems. We investigated the clinical utility of ^18^F-fluorodeoxyglucose positron emission tomography/computed tomography (^18^F-FDG PET/CT) to detect rejection.

**Methods:**

This retrospective single–center study reviewed the clinical data of heart-transplant recipients at our institution between 2008 and 2022. Patients who underwent ^18^F-FDG PET/CT scans post-transplant were enrolled. Scans were performed following a high-fat, low-carbohydrate diet initiated 24 hours before imaging and an 18-hour fasting period. FDG uptake was represented as cardiac metabolic volume (CMV), maximum standard uptake value (SUVmax), and total lesion glycolysis (TLG).

**Results:**

During the study period, 132 ^18^F-FDG PET/CT scans in 40 heart-transplant recipients were identified. Age at transplant was 53 [43-62] years old, and 29 patients (73%) were male. We found 10 scans at the time of significant ACR. No patients experienced AMR. At the time of ACR, CMV (88.2 [22.5-275.8] vs 0.0 [0.0-7.3] ml, *p* < 0.001), SUVmax (9.21 [6.87-16.0] vs 2.98 [2.49-4.48], *p* < 0.001) and TLG (365.3 [78.3-1,908.7] vs 0.0 [0.0-25.8] g, *p* < 0.001) were significantly higher compared to those without rejection. From baseline to the time of ACR, significant increases of CMV, SUVmax, and TLG (*p* < 0.001 for all) were also found.

**Conclusions:**

FDG uptake was significantly elevated at the time of ACR. ^18^F-FDG PET/CT may represent a promising non–invasive diagnostic adjunct for detecting ACR following heart transplantation.

## Background

Heart-transplant recipients have a significant risk of allograft rejection. The clinical parameters, such as symptoms, laboratory data, electrocardiogram, or echocardiography, are insufficiently sensitive to detect rejection. This necessitates frequent screening with endomyocardial biopsies (EMBs).^1^, ^2^ EMB and pathological diagnosis are the current gold standard for screening acute cellular rejection (ACR) and antibody-mediated rejection (AMR).^3^, ^4^ It is generally a safe procedure with low risk of complications but is associated with patient discomfort, damage to the tricuspid valve, and other rare but life-threatening complications, such as ventricular arrhythmia and myocardial perforation.^5^

Quantifying the accuracy of EMB to diagnose ACR and AMR is difficult. The performance of EMB is negatively affected by (1) the limited number of samples obtained to detect a non–uniform pathological process and (2) significant variability in reporting related to the experience of the attending pathologist and the grade of rejection.^6^, ^7^ Furthermore, biopsy-negative rejection is a well–recognized clinical entity defined by depressed left ventricular function and the absence of pathological and immunohistochemical findings of ACR or AMR on EMB, which appears to respond to the intensification of immunosuppressive treatment.^8^

These limitations of EMB–based rejection surveillance underscore the necessity of novel, noninvasive, and accurate methods to detect rejection.^9^, ^10^ ^18^F-fluorodeoxyglucose positron emission tomography/computed tomography (^18^F-FDG PET/CT) is a non–invasive imaging modality used to detect high metabolic activity in myocardial inflammatory conditions, such as cardiac sarcoidosis, and heart-transplant rejection in animal models and kidney/liver transplant rejection in humans.^11^, ^12^, ^13^, ^14^ It can offer potential advantages over EMB as a screening tool for acute heart-transplant rejection, such as its noninvasiveness, and the ability to evaluate the entire myocardium for signs of inflammation. In this study, we aimed to evaluate the utility of ^18^F-FDG PET/CT to diagnose transplant rejection compared with a pathological and clinical diagnosis of rejection.

## Materials and methods

### Ethical considerations

The study was approved by the Kyushu University Hospital institutional review board (ID: 2113-01).

### Patient population and variables evaluated

The clinical data of heart-transplant recipients who underwent transplantation at our institution between 2008 and 2022 were retrospectively reviewed. Among these patients, those who underwent ^18^F-FDG PET/CT at least once following heart transplantation were enrolled in this study. The clinical data were collected from the electronic medical records.

The clinical characteristics of recipients and donors, and other factors such as ischemic time, donor-recipient sex mismatch, and the risk of cytomegalovirus infection were collected. Immunosuppressive agents, laboratory data, echocardiographic parameters, and hemodynamic parameters were collected at the time of each ^18^F-FDG PET/CT scan.

### ^18^F-FDG PET/CT image acquisition and analysis

At our institute, ^18^F-FDG PET/CT was routinely performed approximately 1 year after heart transplantation and annually thereafter as part of institutional practice in heart-transplant recipients, primarily for screening of post-transplant malignancies, with additional scans performed when allograft rejection was clinically suspected.

All ^18^F-FDG PET/CT scans were performed following a standardized myocardial suppression protocol consisting of a high-fat, low-carbohydrate diet initiated 24 hours before imaging, followed by an 18-hour fasting period.^15^

The administered dose of ^18^F-FDG was 4 MBq/kg. Scans were performed after a standardized uptake period following FDG injection, and acquired using either a Discovery ST Elite scanner (GE Healthcare) or a Biograph Vision scanner (Siemens Healthineers, Erlangen, Germany) during the study period. Throughout the study period, there were no major changes in scanner hardware or reconstruction software that would affect quantitative PET analysis.

FDG uptake in the left ventricle (LV) was quantified using 3 parameters: cardiac metabolic volume (CMV), maximum standard uptake value (SUVmax), and total lesion glycolysis (TLG). Standard uptake value (SUV) was obtained from each pixel as pixel activity (injected dose/body weight). A spherical volume of interest corresponding to the entire LV wall was manually drawn, and FDG uptake except for the LV wall was excluded. SUVmax for the volume of interest was automatically calculated. Based on a commonly used definition in inflammatory cardiac PET studies, an SUV of 3.0 for the LV wall was determined as a low cut-off threshold.^16^, ^17^ The volume for the LV wall with SUV ≥3.0 was then measured as CMV.^18^, ^19^ TLG was calculated by multiplying the CMV by the mean SUV within the CMV, representing an accurate 3-dimensional distribution and intensity of inflammation in the entire heart.^19^

### Diagnosis of rejection

Importantly, ^18^F-FDG PET/CT was not implemented as a substitute for EMB, which remained the reference standard throughout the study period. For pathological diagnosis of rejection, EMB specimens from the right ventricle (RV) were obtained, either at intervals defined by our standardized post–transplant surveillance protocol or in response to clinical signs or symptoms suggestive of acute rejection. The EMBs were performed by experienced clinicians, using the standard Seldinger technique via the jugular or femoral vein. A bioptome was used to obtain at least 3 endomyocardial samples from the RV septum, which were immediately preserved in 10% buffered formalin.

The hematoxylin-eosin staining for histopathological analysis and immunofluorescence staining for C4d were performed. Subsequently, the evaluation and grading were performed by the pathologists for both ACR and AMR according to the criteria of the International Society of Heart and Lung Transplantation.^3^, ^4^ In addition to histopathological evaluation and C4d immunohistochemical staining, donor-specific antibodies were routinely measured during post–transplant follow-up as part of standard surveillance for AMR.

In this study, diagnosis of rejection was based on a combination of clinical and pathological findings. Pathological diagnosis of significant rejection was defined as either ACR with grade 2R or more, or AMR with any grade. Clinical rejection was defined as treated rejection, in which active antirejection therapy was administered by the clinical transplant service (e.g., pulse corticosteroid therapy and/or additional immunosuppressive interventions), as well as sudden death with ACR or AMR confirmed by autopsy. In cases in which ACR was pathologically confirmed, the time interval between ^18^F-FDG PET/CT and EMB was recorded.

### Statistical analysis

Continuous variables were expressed as median [interquartile range]. Categorical variables were presented as count (percent). Normality of continuous variables was assessed using visual inspection of histograms and the Shapiro-Wilk test. Differences in continuous data with normal distribution across groups were compared using unpaired Student’s *t*-tests. Variables with skewed distribution were tested by Mann-Whitney U-tests. Categorical data were compared using a similar approach employing chi-square tests or Fisher’s exact tests, as appropriate. Receiver operating characteristic (ROC) analysis was performed to identify the diagnostic performance of CMV, SUVmax, and TLG. The optimal cut-off values for CMV, SUVmax, and TLG were determined using Youden’s index.

Statistical significance was defined as *p* < 0.05. Statistical analysis was performed using JMP, version 17.0.0 (SAS software). ROC analyses and internal validation using bootstrap resampling were conducted using R (version 4.4.1) with the pROC package.

## Results

### Baseline characteristics

**Figure 1:**
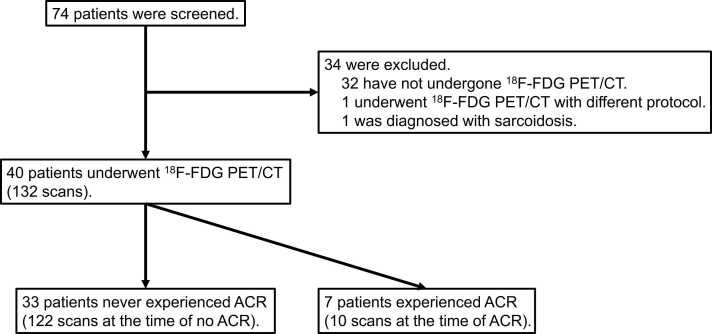
Flowchart of the study. A flowchart of the screening and selection of the patients is shown. ^18^F-FDG PET/CT, ^18^F-fluorodeoxyglucose positron emission tomography/computed tomography; ACR, acute cellular rejection.

Figure 1 represents the flowchart of this study. A total of 74 heart-transplant recipients were screened, among whom 42 underwent at least one ^18^F-FDG PET/CT scan. One patient was excluded from the analysis due to a different imaging protocol, and another was excluded because of suspected recurrence of cardiac sarcoidosis. A total of 132 ^18^F-FDG PET/CT scans were performed on 40 heart-transplant recipients and were analyzed retrospectively. Overall, patients underwent a median of 3 scans (range, 1-9) per patient; 11 patients underwent 1 scan, 8 underwent 2 scans, and 21 underwent 3 or more scans.

Table 1 represents the patient characteristics at the time of transplantation. Median age at transplant was 53 [43-62] years old, and 29 patients (73%) were male. Baseline characteristics of donors and recipients were comparable between the rejection patients and the nonrejection patients.

**Table 1:** Baseline Characteristics of Enrolled Patients

Variables	All patients (*n* = 40)	Nonrejection patients (*n* = 33)	Rejection patients (*n* = 7)	*p*-value
*Recipient characteristics*				
Age, years	53 (43-62)	53 (44-62)	56 (37-63)	0.831
Male sex, *n* (%)	29 (73)	26 (79)	3 (43)	0.338
Asian race, *n* (%)	40 (100)	33 (100)	7 (100)	1.000
Body mass index, kg/m^2^	23.2 (20.6-25.8)	23.2 (20.8-24.8)	26.1 (20.8-27.8)	0.347
Etiology of heart failure, *n* (%)				0.886
Ischemic heart disease	5 (13)	4 (12)	1 (14)	
Dilated cardiomyopathy	24 (60)	21 (64)	3 (43)	
Hypertrophic cardiomyopathy	2 (5)	1 (3)	1 (14)	
Adult congenital heart disease	3 (8)	1 (3)	2 (29)	
Others	6 (15)	6 (18)	0 (0)	
Waiting time before transplantation, days	1,311 (1,062-1,613)	1,370 (1,107-1,610)	1,086 (862-1,511)	0.390
Duration of LVAD support, days	1,402 (1,035-1,594)	1,464 (1,046-1,593)	1,069 (830-1,565)	0.575
*Donor characteristics*				
Age, years	53 (42.8-62.3)	52 (42-62)	60 (51-64)	0.279
Male sex, *n* (%)	24 (69)^a^	20 (69)	4 (67)	0.629
Body mass index, kg/m^2^	22.7 (18.5-25.4)^a^	22.4 (18.6-25.0)	22.9 (17.7-26.7)	0.894
Others				
Allograft ischemic time, minutes	234 (218-260)	237 (220-261)	221 (206-237)	0.303
Donor-recipient sex mismatch, *n* (%)	10 (29)^a^	9 (31)	1 (17)	0.649
CMV high risk, *n* (%)	4 (11)^a^	2 (7)	2 (29)	0.171

Abbreviations: ACR, acute cellular rejection; CMV, cytomegalovirus; LVAD, left ventricular assist device.

Data are expressed as median (interquartile range) or *n* (%).

*p*-values compare baseline characteristics at the time of heart transplantation between recipients who developed ACR during the follow-up period and those who did not.

a Five missing data.

### Clinical characteristics at each ^18^F-FDG PET/CT scan

**Figure 2:**
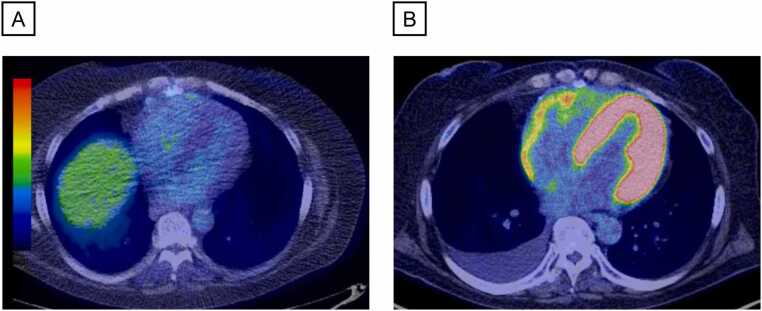
Representative images of ^18^F-FDG PET/CT scan. ^18^F-FDG PET/CT images before the diagnosis of ACR (baseline, panel A) and at the time of ACR (panel B) in the same patient are presented. ^18^F-FDG PET/CT, ^18^F-fluorodeoxyglucose positron emission tomography/computed tomography; ACR, acute cellular rejection.

Figure 2 represents the typical images of ^18^F-FDG PET/CT in the same patient. Compared to the result at baseline (Figure 2A), a diffuse increase of FDG uptake in the LV and RV was seen at the time of ACR (Figure 2B).

Table 2 depicts the summary of clinical characteristics at each ^18^F-FDG PET/CT scan (40 patients, 132 scans). Among the 7 patients who experienced ACR, 3 had 2 separate episodes; therefore, a total of 10 ^18^F-FDG PET/CT scans were obtained at the time of significant ACR. Meanwhile, no patients experienced AMR during the study period. ^18^F-FDG PET/CT scans were performed 1,108 [714-2,191] days following heart transplantation. Serum troponin T (0.061 [0.037-0.091] vs 0.010 [0.000-0.017] ng/ml, *p* = 0.001) and brain natriuretic peptide (251.0 [135.1-310.3] vs 56.0 [29.7-104.8] pg/ml, *p* < 0.001) were significantly higher in the rejection scans. Echocardiography showed that left ventricular end-diastolic diameter and left ventricular end-systolic diameter were significantly higher, whereas left ventricular ejection fraction was significantly lower in the rejection scans (59.4 [52.5-63.1] vs 68.4 [65.1-73.6]%, *p* < 0.001). Hemodynamic data showed that mean pulmonary arterial wedge pressure was significantly higher (13 [10-17] vs 8 [6-11] mm Hg, *p* = 0.023) and cardiac index was significantly lower (2.3 [2.2-2.6] vs 2.8 [2.5-3.3] liters/min/m^2^, *p* = 0.009) in the rejection scans.

**Table 2:** Clinical Characteristics at Each ^18^F-FDG PET/CT Scan (40 Patients, 132 Scans)

Variables	All scans (*n* = 132)	Nonrejection scans (*n* = 122)	Rejection scans (*n* = 10)	*p*-value
Days since heart transplantation, days	1,108 (714-2,191)	1,111 (724-2,223)	1,020 (512-1,449)	0.327
*Laboratory data*				
Troponin T, ng/ml	0.010 (0.000-0.022)	0.010 (0.000-0.017)	0.061 (0.037-0.091)	0.001
BNP, pg/ml	62.1 (30.9-131.8)	56.0 (29.7-104.8)	251.0 (135.1-310.3)	<0.001
CRP, mg/ml	0.07 (0.03-0.32)	0.07 (0.03-0.31)	0.19 (0.04-0.49)	0.435
Creatinine, mg/dl	1.00 (0.87-1.40)	1.00 (0.85-1.37)	1.10 (0.97-1.30)	0.516
BUN, mg/dl	20.0 (15.0-27.3)	19.0 (14.0-25.8)	29.5 (24.5-33.3)	0.002
eGFR, ml/min/1.73 m^2^	60.0 (41.2-74.1)	60.2 (41.1-76.0)	46.5 (44.4-60.5)	0.137
*Echocardiography*				
LVDD, mm	43 (38-46)	43 (37-45)	49 (46-50)	<0.001
LVDS, mm	25 (32-29)	25 (23-28)	31 (29-37)	<0.001
LVEF, %	68.0 (63.6-72.9)	68.4 (65.1-73.6)	59.4 (52.5-63.1)	<0.001
E/A	2.0 (1.6-2.8)	2.0 (1.6-2.7)	2.8 (1.5-3.5)	0.430
E/e'	10.4 (8.2-12.5)	10.1 (8.1-12.4)	11.9 (10.4-12.7)	0.086
Deceleration time, ms	153 (132-179)	153 (133-180)	145 (117-171)	0.582
*Right heart catheterization*				
mPAWP, mm Hg	9 (6-12)	8 (6-11)	13 (10-17)	0.023
CI, liters/min/m^2^	2.8 (2.5-3.3)	2.9 (2.6-3.4)	2.3 (2.2-2.6)	0.009
*Endomyocardial biopsy*				
Histopathological grading of ACR, *n* (%)				0.014
0	2 (2)	2 (2)	0 (0)	
1A (1R)	85 (64)	83 (68)	2 (20)	
1B (1R)	41 (31)	36 (30)	5 (50)	
2 (1R)	2 (2)	0 (0)	2 (20)	
3A (2R)	1 (1)	0 (0)	1 (10)	
N/A	1 (1)	1 (1)	0 (0)	

Abbreviations: ACR, acute cellular rejection; BNP, brain natriuretic peptide; BUN, blood urea nitrogen; CI, cardiac index; CRP, C-reactive protein; eGFR, estimated glomerular filtration rate; LVDD, left ventricular diastolic diameter; LVDS, left ventricular systolic diameter; LVEF, left ventricular ejection fraction; mPAWP, mean pulmonary arterial wedge pressure.

Data are expressed as median (interquartile range) or *n (%).*

Among the 10 rejection scans, ACR was histologically confirmed by EMB in 3 cases (pathological rejection), whereas the remaining 7 cases were clinically diagnosed and treated as rejection despite negative biopsy findings (clinical rejection). In the pathological rejection cases, the median time interval between ^18^F-FDG PET/CT and EMB was 4.5 [2.0-14.3] days. Detailed PET parameters stratified by the pathological rejection, clinical rejection, and nonrejection scans were provided in Supplementary Table S1. Of these 10 rejection scans, 6 were performed as part of routine surveillance follow-up, whereas 4 were obtained in the setting of clinical suspicion due to new symptoms or clinical deterioration.

**Figure 3:**
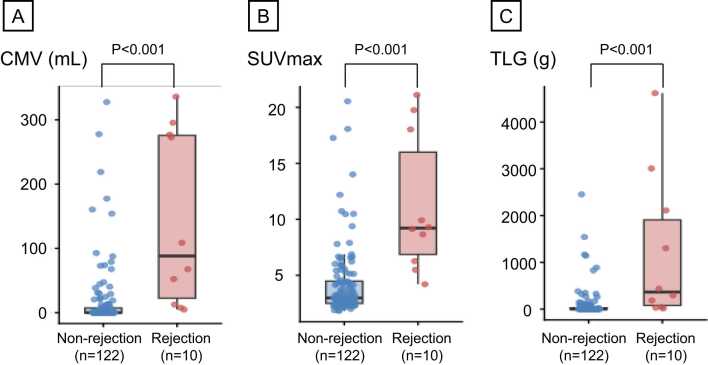
Comparison of CMV, SUVmax, and TLG between the rejection and nonrejection scans. CMV (Panel A), SUVmax (Panel B), and TLG (Panel C) of ^18^F-FDG PET/CT scans in the rejection and nonrejection scans are depicted. CMV, cardiac metabolic volume; SUVmax, maximum standard uptake value; TLG, total lesion glycolysis.

Figure 3 represents the comparison of ^18^F-FDG PET/CT parameters between the rejection and the nonrejection scans. CMV (88.2 [22.5-275.8] vs 0.0 [0.0-7.3] ml, *p* < 0.001, Figure 3A), SUVmax (9.21 [6.87-16.0] vs 2.98 [2.49-4.48], *p* < 0.001, Figure 3B), and TLG (365.3 [78.3-1,908.7] vs 0.0 [0.0-25.8] g, *p* < 0.001, Figure 3C) in the rejection scans were significantly higher compared to those in the nonrejection scans.

**Figure 4:**
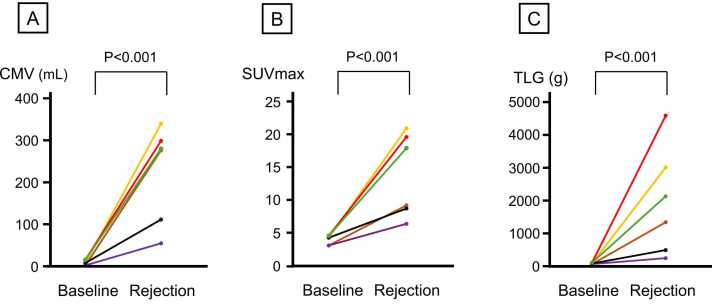
Consecutive changes of CMV, SUVmax, and TLG from baseline to ACR. The changes of CMV (A), SUVmax (B), and TLG (C) in the same patients before the diagnosis of ACR (baseline) to those at the time of ACR are shown. CMV, cardiac metabolic volume; SUVmax, maximum standard uptake value; TLG, total lesion glycolysis.

Consecutive ^18^F-FDG PET/CT scans at baseline (before the time of ACR) and at the time of ACR were found in 6 episodes. Significant increase of CMV (5.7 [1.4-11.5] vs 274.8 [149.7-290.8] ml, *p* < 0.001, Figure 4A), SUVmax (4.11 [3.21-4.33] vs 13.58 [8.77-19.32], *p* < 0.001, Figure 4B), and TLG (18.4 [18.4-45.3] vs 2,110.6 [1,302.9-3,008.3] g, *p* < 0.001, Figure 4C) were found in these patients.

### Utility of ^18^F-FDG PET/CT scans for detecting ACR

**Figure 5:**
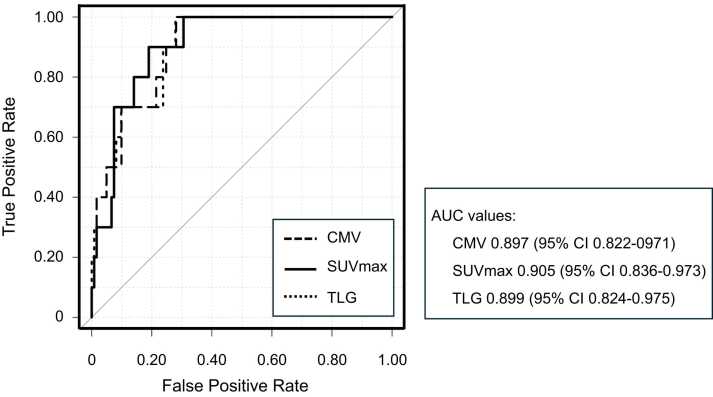
ROC curves of CMV, SUVmax, and TLG for diagnosing ACR. The ROC analyses for diagnosing ACR using CMV, SUVmax, and TLG are shown. AUC values of these parameters are also shown. AUC, area under the curve; CI, confidence interval; CMV, cardiac metabolic volume; SUVmax, maximum standard uptake value; TLG, total lesion glycolysis.

Figure 5 demonstrates the ROC curves based on CMV, SUVmax, and TLG, which were drawn for detecting ACR. The utility of CMV, SUVmax, and TLG for diagnosing ACR was evaluated as area under the curve (AUC) of 0.897 (95% confidence interval 0.822-0.971), 0.905 (0.836-0.973), and 0.899 (0.824-0.975), respectively. The sensitivity and specificity were 100% (69-100) and 72% (63-80) for CMV (cut-off value: 4.37 ml), 90% (55-100) and 81% (73-88) for SUVmax (cut-off value: 5.48), and 100% (69-100) and 72% (63-80) for TLG (cut-off value: 14.50 g), respectively. Given the limited number of ACR events, we further performed internal validation using bootstrap resampling (2,000 iterations) to assess the robustness of the ROC analyses. Bootstrap analysis demonstrated stable AUC estimates for CMV (0.897, 95% confidence interval 0.812-0.963), SUVmax (0.905, 0.825-0.967), and TLG (0.899, 0.815-0.968), supporting the robustness of the ROC analyses.

## Discussion

Although EMB and pathological diagnosis are the current gold standard for screening myocardial rejection, alternative noninvasive and accurate diagnostic tools are desired instead of EMB owing to its potential procedural risks and insufficient diagnostic performance.^10^, ^20^ Previous studies have reported the feasibility of ^18^F-FDG PET in transplant rejection models or other organ transplantation.^11^, ^13^, ^14^, ^21^ Furthermore, recent human studies have reported the feasibility of ^18^F-FDG PET/CT for detecting cardiac allograft rejection, including qualitative myocardial FDG uptake in patients with definite or suspected rejection.^22^ The present study extends the existing literature by providing serial quantitative PET metrics (CMV, SUVmax, and TLG) across a broader clinical spectrum, including surveillance scans without clinical suspicion of rejection, and by evaluating diagnostic performance using ROC analyses.

### The importance of alternative diagnostic tools for myocardial rejection

The evaluation of clinical rejection has continued to rely on repetitive invasive procedures, namely the histologic evaluation of EMB specimens to diagnose both ACR and AMR. The early diagnosis and treatment of myocardial rejection are a major pillar in the management of heart-transplant recipients. The goal is to preserve graft function, extend patient survival, and avoid cardiac allograft vasculopathy, which is a major cause of long-term allograft failure and mortality, and is at least partly related to rejection.^23^ As clinical parameters such as symptoms, laboratory data, electrocardiogram, and echocardiography are insufficiently sensitive to detect heart-transplant rejection, EMB has remained a hallmark of post-transplant care despite its risk of complications and lack of reproducibility in the diagnosis of rejection.^1^, ^8^, ^24^ Reliable noninvasive tools to detect myocardial rejection before the development of graft dysfunction would possibly result in better outcomes for those patients who develop allograft rejection.

Recent research indicates that molecular biomarkers such as gene-expression profiling and donor–derived cell-free DNA are emerging as alternative tools to EMB, often in combination with imaging modalities such as cardiac magnetic resonance (CMR).^25^, ^26^, ^27^, ^28^, ^29^ The present study investigated the utility of non–invasive ^18^F-FDG PET/CT imaging, where the utility to detect high metabolic activity has been established in myocardial inflammatory conditions such as cardiac sarcoidosis and myocarditis.^12^, ^30^ Given that myocardial rejection represents an inflammatory process, it is biologically plausible that ^18^F-FDG PET/CT can detect myocardial rejection.

CMR imaging and ^18^F-FDG PET/CT provide complementary information in the evaluation of cardiac allograft rejection. CMR primarily offers structural and tissue-characterization insights, such as myocardial edema, fibrosis, and extracellular volume expansion, which reflect downstream consequences of myocardial injury. In contrast, ^18^F-FDG PET/CT assesses active inflammatory metabolism and may therefore capture ongoing immune–mediated myocardial inflammation at an earlier or more dynamic stage.

The rapid expansion of noninvasive modalities has resulted in a lack of clear clinical guidance on how to best incorporate them into post-transplant care. ¹⁸F-FDG PET/CT may complement emerging noninvasive strategies, including donor–derived cell-free DNA and gene-expression profiling, by providing spatial and functional information on myocardial inflammatory activity that blood-based biomarkers cannot localize. Rather than replacing EMB or CMR, ^18^F-FDG PET/CT is more likely to serve as a complementary diagnostic adjunct in selected and complex cases of suspected rejection.^10^, ^20^ The potential of ^18^F-FDG PET/CT to reduce unnecessary EMBs in selected patients should be validated in larger prospective studies and cannot be concluded from the present data.

### Mechanisms of increased glucose metabolism

The uptake of FDG represents glucose metabolism in the myocardium, and its possibility of diagnostic performance for acute rejection has been demonstrated in mouse models and in human acute renal rejection.^11^, ^14^

It has also been reported that increased uptake of FDG can be seen in patients with myocarditis, and that ^18^F-FDG PET/CT was useful for its diagnosis.^30^ The pathophysiology of ACR is similar to acute lymphocytic myocarditis, which is mainly caused by T cell–mediated inflammatory response in the myocardium. Lymphocytic infiltration is also observed in cardiac sarcoidosis, where the diagnostic importance of ^18^F-FDG PET/CT has been established.^12^, ^31^ We hypothesized that the possible mechanisms of increased glucose metabolism in the myocardium are as follows: (1) glucose metabolism of infiltrated lymphocytes, and (2) alteration of metabolism from fatty acid to glucose in the injured cardiomyocytes.

In this study, we did not observe any cases of AMR and were thus unable to evaluate the potential role of ^18^F-FDG PET/CT in its diagnosis. Moreover, there are currently no established reports demonstrating the diagnostic utility of ^18^F-FDG PET/CT for AMR in heart transplantation.

### Clinical implications

As mentioned above, the limitations of EMB diagnosis have motivated us to investigate an alternative approach to the diagnosis of myocardial rejection.^5^, ^6^, ^8^, ^9^ The current alternative modalities proposed include gene-expression profile, donor–derived cell-free DNA, and imaging modalities such as CMR.^25^, ^26^, ^27^, ^28^, ^29^ This study clearly demonstrated that ^18^F-FDG PET/CT may represent a promising noninvasive adjunct with high sensitivity for detecting ACR. In contrast, the specificity of ^18^F-FDG PET/CT was relatively low in this study, suggesting residual physiological uptake of FDG in the myocardium.^32^ The low specificity of ^18^F-FDG PET/CT is also reported in the diagnosis of cardiac sarcoidosis.^19^ The feasibility of our myocardial suppression protocol, consisting of a high-fat dietary preparation and prolonged fasting, should be further validated and optimized in future studies.

### Limitations

This study has several limitations. First, it is a single-center study with a retrospective design and a relatively small number of enrolled patients. Especially, due to the small number of the rejection group, it was difficult to evaluate the relationship between the findings of ^18^F-FDG PET/CT and the severity of ACR. The utility of ^18^F-FDG PET/CT should be validated in multicenter and prospective studies. The small number of the rejection group may also have resulted in statistical fragility and potentially optimistic estimates of diagnostic performance in ROC analyses. Although internal validation was performed using bootstrap resampling, these estimates remain subject to sampling variability. In addition, multiple rejection scans were obtained from the same patients (10 scans in 7 patients) and analyzed as independent observations, which may not fully account for within-patient clustering and may have led to underestimation of variance and inflation of statistical significance, including *p*-values and AUC estimates. Moreover, CMV was defined using an SUV threshold of 3.0 based on prior inflammatory cardiac ^18^F-FDG PET/CT studies^16^; however, the optimal SUV threshold in denervated transplanted hearts remains uncertain. Therefore, the diagnostic performance and cut-off values derived from CMV, SUVmax, and TLG should be interpreted as exploratory and hypothesis-generating rather than definitive for clinical implementation. Second, the diagnosis of myocardial rejection was determined by attending cardiologists based on pathological and clinical findings. Due to the limited accuracy of pathological diagnosis, previous studies have also defined the diagnosis of myocardial rejection in combined multiple modalities. The results of ^18^F-FDG PET/CT were not blinded to the treating cardiologists and were available at the time clinical decisions were made. Although clinical decisions were not directly driven by ^18^F-FDG PET/CT findings, the availability of ^18^F-FDG PET/CT findings may have influenced clinical judgment to some extent. Third, this study included both ^18^F-FDG PET/CT scans for surveillance and at the time of suspected rejection. Pretest probabilities of these scans must be different and may have affected the interpretation of the results. Although annual ^18^F-FDG PET/CT surveillance was implemented as an institutional practice, some patients did not undergo it due to several reasons. Fourth, no cases of AMR were observed during the study period. Although EMB specimens were routinely evaluated for AMR, including immunohistochemical staining for C4d, the absence of AMR cases precluded any assessment of the diagnostic utility of ^18^F-FDG PET/CT for AMR. Finally, follow-up data after the diagnosis of rejection were not available, and therefore, it was difficult to evaluate the ability of ^18^F-FDG PET/CT to assess treatment response and longitudinal outcome data, such as graft survival and the progression of cardiac allograft vasculopathy.

## Conclusion

FDG uptake in the LV was significantly elevated at the time of ACR. ^18^F-FDG PET/CT may represent a promising non–invasive diagnostic adjunct for detecting ACR following heart transplantation.

## Funding

This research was supported by JSPS KAKENHI Grant No. JP22K08103 (T.F.) and JSPS KAKENHI Grant No. JP25K11412 (T.F.).

## CRediT authorship contribution statement

T.Y. and T.F. contributed to the conceptualization and methodology of this study. T.Y., T.F., T.H., K.M., Y.K., T.I., and S.B. contributed to the investigation of this study. T.Y., S.Y., K.I., T.S., K.S., and S.M. contributed to the formal analysis of this study. T.Y. and T.F. contributed to the writing (original draft) of the manuscript. All authors contributed to the writing (review and editing) of the manuscript. S.K., K.I., A.S., and K.A. contributed to the supervision of this study. T.F. contributed to the funding acquisition of this study.

## Declaration of Competing Interest

The authors declare the following financial interests/personal relationships which may be considered as potential competing interests: Takeo Fujino, a member of the endowment department supported by Abbott Medical Japan, Medtronic, and NIPRO Corporation. The other authors declare that they have no known competing financial interests or personal relationships that could have appeared to influence the work reported in this paper.

## Data Availability

The datasets used and/or analyzed in this study are available from the corresponding author on reasonable request.

## Footnotes

Appendix A Supplementary data associated with this article can be found in the online version at doi:10.1016/j.jhlto.2026.100526.
