# Supplementary material for: 18F-Fluorodeoxyglucose positron emission tomography for detection of acute cellular rejection after heart transplantation: A single–center retrospective study
Source: JHLT Open. 2026 Feb 24;12:100526. doi: 10.1016/j.jhlto.2026.100526 (PMC13010973; doi:10.1016/j.jhlto.2026.100526)
Supplement: Supplementary file 1 — Supplementary material [file mmc1.docx]

Supplementary Table S1. PET parameters stratified by the pathological rejection, clinical rejection, and non-rejection scans.

|  | Pathological rejection  (ACR ≥2R or any AMR) | Clinical rejection | Non-rejection |
| --- | --- | --- | --- |
| n (scans) | 3 | 7 | 122 |
| CMV, mL | 272.6 (67.7–276.7) | 52.2 (7.6–295.4) | 0.0 (0.0–7.3) |
| SUVmax | 9.30 (9.12–18.03) | 8.7 (5.5–19.8) | 2.98 (2.49–4.48) |
| TLG, g | 1302.9 (295.0–2110.1) | 185.8 (29.5–3008.8) | 0.0 (0.0–25.8) |

ACR, acute cellular rejection; AMR, antibody-mediated rejection; CMV, cardiac metabolic volume; SUVmax, maximum standard uptake value; TLG, total lesion glycolysis.
